# Effects of shoe weight on landing impact and side-to-side asymmetry

**DOI:** 10.1371/journal.pone.0256061

**Published:** 2021-08-12

**Authors:** I-Lin Wang, Jin-Jiang Gao, Li-I Wang, Ke-Ke Zhang

**Affiliations:** 1 College of Physical Education, Hubei Normal University, Huangshi, Hubei, China; 2 Department of Physical Education and Kinesiology, National Dong Hwa University, Hualien, Taiwan, R.O.C; 3 Graduate Institute, Jilin Sport University, Changchun, Jilin, China; University of L’Aquila, ITALY

## Abstract

Shoes of different weights affect proprioception. Drop jump (DJ) tasks are often used to explore the risks and mechanisms of lower limb injuries. Leg dominance mainly refers to differences between the dominant and nondominant legs. Eight males and eight females participated in this study. The weights of the shoes in this investigation were 255 g, 335 g, and 415 g, and the heights of the DJ were 30 cm, 40 cm, and 50 cm. The side-to-side asymmetry of the time of contact initiation for the 30DJ was greater than that of the 40DJ and 50DJ, and the asymmetry for shoes weighing 415 g and 355 g was greater than that for shoes weighing 255 g. When the drop height increased, the side-to-side asymmetry of the peak ground reaction force (PGRF). also increased. The ground contact time increased as the drop height increased to 50DJ. Higher drop heights caused greater side-to-side asymmetry of the PGRF. Heavier shoes caused the peak ground reaction force time (PGRFT) in the nondominant leg to appear earlier, reduced the jump height and affected the performance. Heavier shoes caused greater side-to-side asymmetry at the initial ground contact and at the ground contact time, affecting training effectiveness.

## Introduction

Generally, sports shoes are now designed to be lightweight because it is believed that light shoes can enhance athletic performance. In a previous study, the shoes with a lighter weight led to better shuffle-cut and vertical-jump performance when the participants were informed about the weight differences between shoe conditions before the test [1]. Weight differences among shoes are attributed to the different materials used and differences in the texture of the soles; past research has shown a dancer’s level of proprioception can differ when he or she is barefoot and wearing ballet shoes with different textures [2]. Previous research has also shown that running shoes of different weights can affect running economy. For example, individuals expend more energy when running in rocker shoes than when running in standard running shoes or minimalist shoes because of the larger shoe mass [3]. Additionally, the weight of shoes can affect muscle activity levels during running [4]. Previous studies have examined the designs of running shoes and assessed shoes of different weights (minimalist shoes, approximately 200 g; tracker shoes, 400 g); the results showed that shoe weight may be the main factor affecting running stride.

Damage to the lower extremities usually occurs when the speed of movement increases or the direction of motion changes, such as during drop landing (DL), drop jump (DJ) and stop jump tasks, especially when the tibia is in the internal rotation position and the knee joint is nearly straight [5]. Therefore, relevant studies often use DL [6, 7], DJ [8, 9], and stop jump [10] tasks to explore the risks and mechanisms of lower limb injuries. Hewett, Ford [11] found that overexposure of the knee valgus causes ligament tearing. During the DJ task, the PGRF, PGRFT and loading rate (LR) are related to the risk of lower limb injury [12].

Leg dominance mainly refers to the differences between the dominant and nondominant legs in factors including muscle strength and muscle stiffness and the imbalance between muscle strength and recruitment patterns. The dominant leg exhibits higher strength and coordination abilities than does the non-dominant leg, which can lead to a greater leg and vertical stiffness [13]. Individuals tend to have greater dynamic control on the side of the dominant leg. Excessive reliance on one side can cause additional stress on the knee joint, and the nondominant leg may not effectively absorb the impact. Therefore, biomechanical studies have explored the association between side-to-side asymmetry of the feet and injury based on this theory [11]. Side-to-side asymmetry of the knee valgus angle during the landing phase will result in greater strength and force of the dominant leg, and greater side-to-side asymmetry of the strength and moment of force between the legs will result in a greater risk of ACL injury [14, 15]. Previous studies that have compared individuals with and without ACL injuries have used a 31-cm vertical DJ task to assess female athletes. During the landing phase, the angle of the knee valgus in both legs was significantly different between the groups with and without ACL injury. The side-to-side asymmetry of the knee joint movement in the ACL injury group was significantly greater than that in the uninjured group. Therefore, the side-to-side asymmetry of the knee valgus moment can be used to predict ACL injury [15].

For DJ tasks from the same height, the PGRF of the dominant leg will be greater than that of the nondominant leg. This shows that the dominant leg has a higher force and moment of force; this may occur because the subject is overly dependent on the dominant leg, which increases pressure on the knee joint. The nondominant ankle has a more effective protective mechanism in that excessive joint motion is restrained by greater ankle flexor activity, and the dominant ankle joint is at greater injury risk during DJ [13]. In summary, the weights of the various types of shoes worn by participants are very different, and whether different shoe weights affect the biomechanical properties of the jumping movement is unknown. This study aimed to investigate the effect of different shoe weights on PGRF characteristics and injury risk during the landing stage. It was hypothesized that a greater shoe mass would result in an increased side-to-side asymmetry PGRF during a DJ.

## Materials & methods

### Participants

Eight male and eight female physical education students (21.7 ± 1.9 years, 1.66 ± 0.08 m, and 59.19 ± 9.36 kg) volunteered for this study and provided written informed consent. None of the subjects had a history of lower extremity injuries during the six months prior to the experiment, and all were participating in sports a minimum of four times per week on average. The subjects were already familiar with shoes of different weights. The study was approved by the Antai Medical Care Corporation Memorial Hospital (No. 15-066-B1).

### Procedures

During data collection, all participants wore identical running shoes (brand: NBU; model: NS-2016001) to reduce variability. Four of the same shoes with different weights were used in this study, and lead weights were uniformly distributed over the four outer sides of each shoe to meet the total weight required. The testing conditions were as follows balanced sequence: shoe only (175 g +/− 5 g), shoe weight plus with an additional 4 × 20 g lead weight (255 +/− 5 g), shoe weight plus with an additional 4 × 40 g lead weight (335 +/− 5 g), and shoe weight plus with an additional 4 × 60 g lead weight (415 +/− 5 g). The DJ task was performed at three heights, namely, 30, 40 and 50 cm (DJH30, DJH40 and DJH50, respectively), and the trial height order was randomized for each participant. The subjects were instructed to warm up by stretching for 20 minutes and to practice the jump three times at each shoe weight before data collection to familiarize themselves with the procedure. The subjects performed each jump with their hands on their waist, and the subjects were required to land simultaneously with both feet onto 2 force platforms. The subjects were asked to spend as little time possible in contact with the ground and to rebound as quickly as possible, jumping to the maximal height [16]. Each participant was asked to perform three successful trials for each drop height and each shoe weight. And all participants rested for 15 minutes between each drop height and shoe weight condition to avoid fatigue interfering with the experiment.

### Processing

The subjects were instrumented with 40 retro-reflective markers for 3D biomechanical analysis. The markers were arranged in a modified Helen Hayes format, as previously described [4]. Motion data were collected with an 8-camera motion analysis system (Qualisys Track Manager [QTM], Oqus 100, Sweden) with a sampling rate of 200 Hz. Two force platforms (BP600900, AMTI Inc., Watertown, MA, USA) with a sampling rate of 1000 Hz were used to record the GRFs for each foot. The kinematic and kinetic data were synchronized with a Qualisys 64-channel interface. To estimate the moment of initial contact, the kinetic parameters were used to determine the gait phase. The time at which the vertical ground reaction force value reached 10 N was defined as the initial ground contact time. The 3D coordinates of the reflective markers and the force data were imported into MATLAB software (version 7.0; The MathWorks Inc., Natick, MA) from the QTM software for data reduction and analysis. The kinematics data were low-pass filtered using a fourth-order Butterworth filter with a cut-off frequency of 12 Hz, and the kinetics data were low-pass filtered using a fourth-order Butterworth filter with a cut-off frequency of 50 Hz. Only the first contact phase of the vertical DJ was analyzed. The flight phase of the DJ was defined as the phase from foot take-off from the force platform to foot contact during the second landing on the force platform. The contact phase was identified by the GRF data. Ground contact time refers to the time when the foot is in contact with the platform. Initial contact time was defined as the moment when the vertical ground reaction force exceeds 10 N. PGRFT was defined initial contact time to the PGRF during the first drop jump landing. LR was calculated by dividing the maximal vertical force by the time to the maximal vertical force [17]. The LR was determined by normalizing PGRF to body mass (BW) and the duration. The jumping height (JH) was calculated using the following formula: JH = gT (flight phase)^2^/8. The flight phase defined the total force (right ground force add left ground force) that take off time to second landing time during the DJ with two feet in per trial. Side-to-side asymmetry was calculated using the following formula: |value of dominant leg–value of nondominant leg| [18]. The dominant leg was examined by kicking preference and defined on the right side [19].

### Statistical analyses

The data were analyzed using the Statistical Package for the Social Sciences (SPSS) 14.0 for Windows. Two-way repeated measures ANOVA was used to determine the main effects of and interactions between DJ heights of 30, 40 and 50 cm (DJH30, DJH40 and DJH50, respectively) and shoe weights of 255, 355 and 415 g (S255, S335, and S415, respectively) on the PGRFT, PGRF, LR and ground contact time. In the presence of a significant interaction effect between DJ height and shoe weight, the least significant difference (LSD) method (*p* < 0.05) was used to compare DJH30, DJH40 and DJH50 independently with S255, S335, and S415. If a significant interaction did not exist, the main effects between shoe weights or DJ heights were analyzed. The significance level was set at *p* = 0.05.

## Results

Table 1 summarizes the DJ performance and the kinetics of the interaction between the DJ height and shoe weight. The p-values were greater than 0.05 (*p* > 0.05) for the interaction between DJ height and shoe weight for PGRFT, PGRF, LR and ground contact time in the dominant and nondominant legs. But the dominant leg and nondominant PGRFT, PGRF, LR and ground contact time values were significantly affected by DJ height. The PGRFT of the nondominant leg was significantly affected by shoe weight (S255 = 88.35 ± 0.83 ms, S335 = 72.44 ± 7.61 ms, S415 = 69.66 ± 5.99 ms, respectively, *p* = 0.044). The post hoc results were significant for S255 and S335 (P = 0.004).

**Table 1:** Dominant leg and nondominant leg: Mean (SD) with the different shoe weights during DJs from 3 heights.

Leg			DJH_30_	DJH_40_	DJH_50_	Interaction
						p-value
**Dominant leg**	PGRFT (ms) ^H^	S_255_	106.40 ± 68.51	83.27 ± 51.74	65.07 ± 21.49	0.343
		S_335_	92.18 ± 66.85	66.71 ± 26.07	55.27 ± 12.09	
		S_415_	83.54 ± 40.22	63.98 ± 17.85	63.01 ± 17.17	
	PGRF (BW) ^H^	S_255_	1.80 ± 0.52	2.24 ± 0.56	2.53 ± 0.71	0.488
		S_335_	1.83 ± 0.57	2.30 ± 0.67	2.63 ± 0.89	
		S_415_	1.61 ± 0.52	2.23 ± 0.61	2.51 ± 0.65	
	LR (BW/ms) ^H^	S_255_	27.82 ± 19.79	37.64 ± 18.95	46.24 ± 21.11	0.725
		S_335_	33.05 ± 27.37	48.18 ± 30.80	54.34 ± 31.28	
		S_415_	27.06 ± 16.47	38.99 ± 14.38	44.81 ± 17.97	
	Ground contact time (ms) ^H^	S_255_	471.50 ± 98.51	460.11 ± 119.42	500.11 ± 79.76	0.223
		S_335_	469.16 ±100.80	497.03 ± 88.55	493.99 ± 82.92	
		S_415_	476.66 ± 91.74	498.10 ± 136.37	505.79 ±110.21	
**Nondominant leg**						
	PGRFT (ms) ^H,S^	S_255_	107.86 ± 72.09	90.28 ± 49.67	66.90 ± 22.66	0.145
		S_335_	92.88 ± 63.42	65.75 ± 24.72	58.70 ± 12.01	
		S_415_	79.71 ± 41.72	64.06 ± 19.11	65.20 ± 17.09	
	PGRF (BW) ^H^	S_255_	1.49 ± 0.34	1.95 ± 0.40	2.26 ± 0.54	0.724
		S_335_	1.44 ± 0.35	1.88 ± 0.50	2.33 ± 0.49	
		S_415_	1.43 ± 0.34	1.88 ± 0.56	2.26 ± 0.53	
	LR (BW/ms) ^H^	S_255_	22.24 ± 16.61	30.32 ± 14.63	39.24 ± 15.45	0.207
		S_335_	25.80 ± 24.11	54.30 ± 66.02	45.19 ± 24.41	
		S_415_	31.17 ± 20.18	32.29 ± 12.35	38.73 ± 15.26	
	Ground contact time (ms)^H^	S_255_	465.48 ± 97.91	456.82 ± 122.04	499.74 ± 80.87	0.283
		S_335_	462.65 ±100.59	488.74 ± 86.52	490.29 ± 82.56	
		S_415_	469.69 ± 88.59	490.42 ± 133.38	503.04 ± 98.02	

H indicates a significant difference in the main effect of DJ height (p<0.05).

S indicates a significant difference in the main effect of shoe weight (p<0.05).

**Fig 1:**
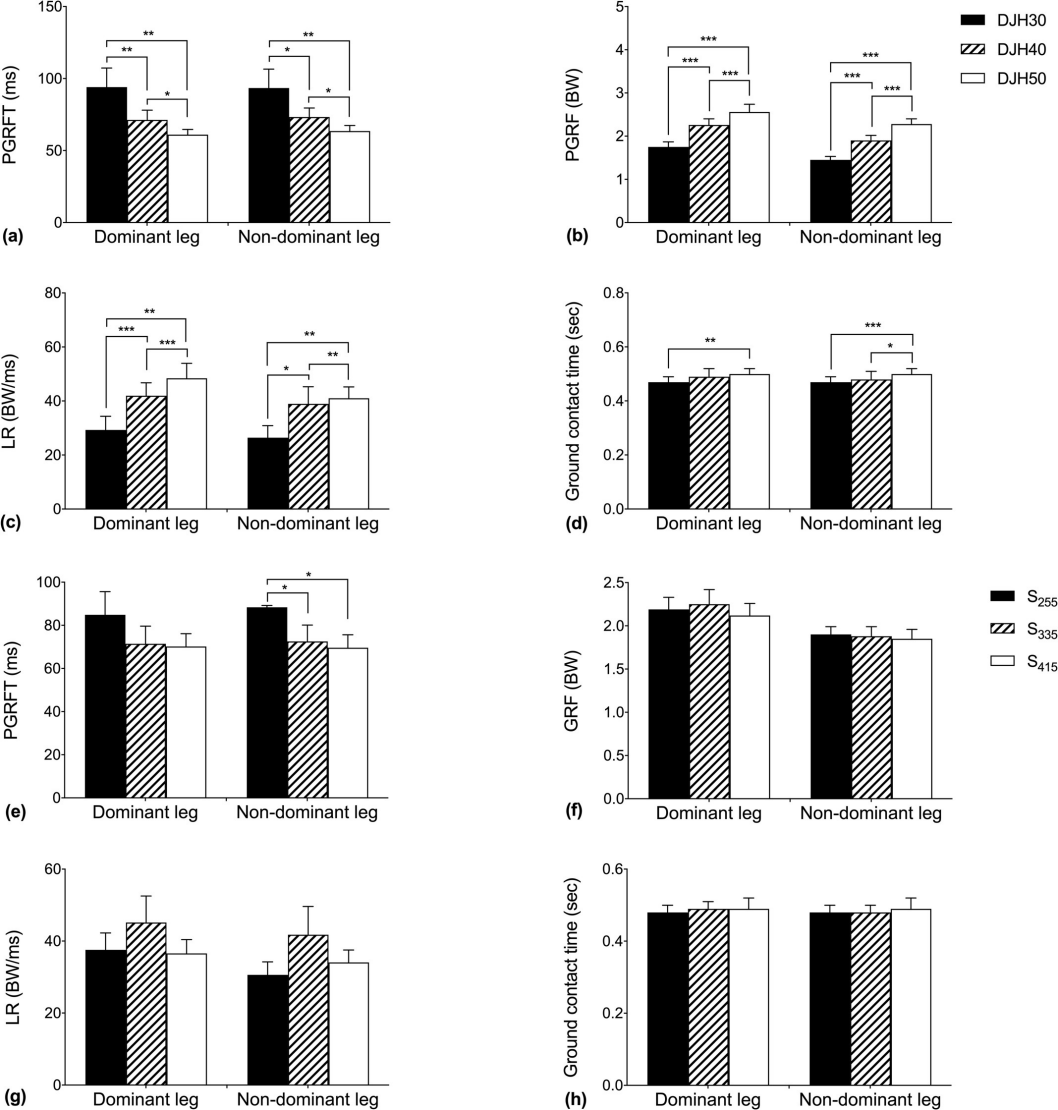
Dominant leg and nondominant leg in the mean (SD) main effects of jumping heights and shoes weights. *Indicates a significant difference from the drop height (p<0.05); **Indicates a significant difference from the drop height (p<0.01); ***Indicates a significant difference from the drop height (p<0.001).

Fig 1 summarizes the results of the dominant and nondominant legs for the main effect of drop height and shoe weight. The values of the PGRFT gradually decreased at DJH30, DJH40 and DJH50 for the dominant and nondominant legs (Fig 1A, *p* = 0.006, 0.011). The post hoc results were significant for H30 and H40 (*p* = 0.010), H30 and H50 (*p* = 0.007), H40 and H50 (*p* = 0.038) in dominant legs. The post hoc results were significant for H30 and H40 (*p* = 0.035), H30 and H50 (*p* = 0.007), H40 and H50 (*p* = 0.018) in nondominant legs. The PGRF, LR and ground contact time (Fig 1B–1D) increased as the drop height increased at DJH_30_, DJH_40_ and DJH_50_. The PGRFT (Fig 1E) in the nondominant leg was mainly affected by shoe weight (S_255_ more than the S_335_ and S_415_).

Table 2 summarizes the jump height and side-to-side asymmetry of leg kinetics parameters of the interaction between the drop height and the shoe weight. There were no significant interactions between DJ height and shoe weight. The jump height, PGRFT, initial contact time and ground contact time showed “main effect” with DJ height and shoe weight.

**Table 2:** The jump height and side-to-side asymmetries of leg kinetics: Mean (SD) with the different shoe weights during DJs from 3 heights.

		DJH_30_	DJH_40_	DJH_50_	Interaction
					p-value
**Jump height (m)** ^***S***^	S_255_	0.29 ± 0.08	0.29 ± 0.07	0.29 ± 0.07	0.082
	S_335_	0.28 ± 0.07	0.28 ± 0.06	0.28 ± 0.06	
	S_415_	0.27 ± 0.07	0.27 ± 0.06	0.28 ± 0.07	
**PGRFT (ms)** ^***H***^	S_255_	0.42 ± 0.36	0.40 ± 0.23	0.51 ± 0.33	0.400
	S_335_	0.49 ± 0.38	0.63 ± 0.37	0.60 ± 0.48	
	S_415_	0.38 ± 0.23	0.54 ± 0.42	0.56 ± 0.35	
**Initial ground contact (ms)** ^***H、S***^	S_255_	7.87 ± 5.41	5.44 ± 3.89	4.16 ± 1.73	0.734
	S_335_	9.04 ± 5.67	8.21 ± 4.67	6.01 ± 3.18	
	S_415_	9.69 ± 5.76	8.21 ± 5.64	4.97 ± 2.58	
**Ground contact time (ms)** ^***H、S***^	S_255_	9.69 ± 4.52	7.21 ± 3.94	6.00 ± 2.55	0.320
	S_335_	9.65 ± 6.30	10.67 ± 5.90	9.08 ± 5.58	
	S_415_	10.85 ± 6.72	11.88 ± 4.59	8.25 ± 3.96	

H indicates a significant difference in the main effect of DJ height (p<0.05).

S indicates a significant difference in the main effect of shoe weight (p<0.05).

**Fig 2:**
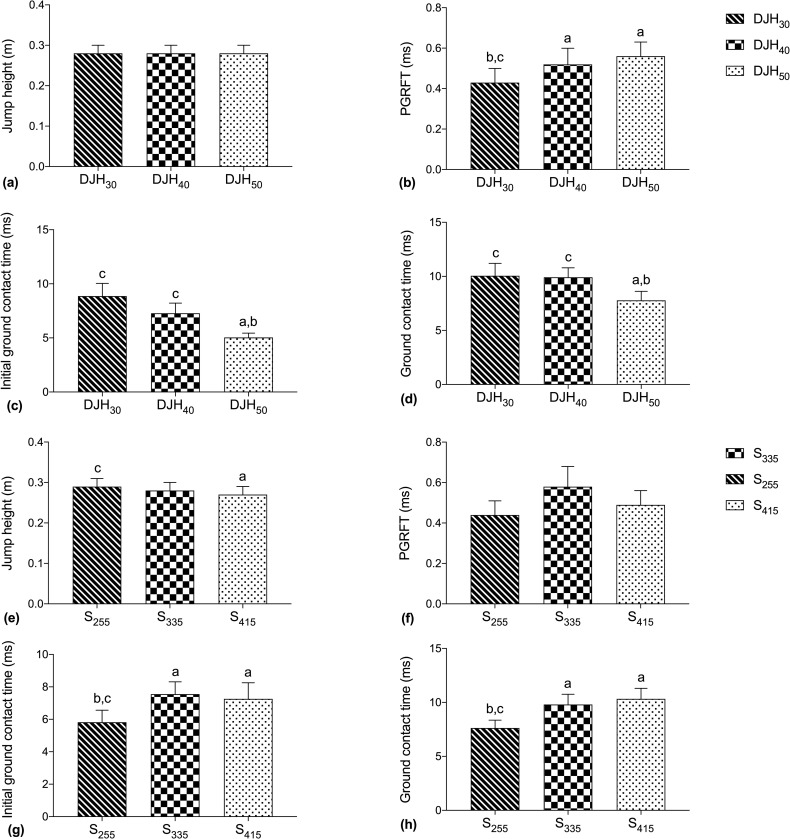
The side-to-side asymmetries in leg kinetics: mean (SD) main effects of drop height (a)~(d) and shoe weight (e)~(h). For the main effects of drop height, a: indicates a significant difference from DJH_30_; b: indicates a significant difference from DJH_40_, c: indicates a significant difference from DJH_50_; for the main effects of shoe weight, a: indicates a significant difference from S_225_; b: indicates a significant difference from S_335_, and c: indicates a significant difference from S_415_.

Fig 2 shows that the side-to-side asymmetry difference for the main effect of drop height and shoe weight. The values of the PGRFT differed by drop height gradually increased at DJH_30_, DJH_40_ and DJH_50_ (Fig 2B, *p* = 0.001). The post hoc results were significant for H30 and H40 (*p* = 0.025), H30 and H50 (*p* = 0.001). The initial ground contact time values for the different drop heights gradually decreased at DJH_30_, DJH_40_ and DJH_50_ (Fig 2C, *p* <0.001). The post hoc results were significant for H30 and H50 (*p* = 0.001). The initial ground contact time for different shoe weights was S_255_ less than S_335_ and S_415_ (Fig 2G, *p* = 0.001)_._ The post hoc results were significant for S_255_ and S_335_ (*p* <0.001), S_255_ and S_415_ (*p* = 0.024). The side-to-side asymmetry of JH for different shoe weights exhibited S_255_ more than S_415_ (Fig 2E, *p* = 0.002). The values for ground contact time differed by drop height gradually decreased at DJH_30_, DJH_40_ and DJH_50_ (Fig 2D, *p* = 0.033). The post hoc results were significant for H30 and H50 (*p* = 0.019). The ground contact time for different shoe weights exhibited S_255_ less than S_335_ and S_415_ (Fig 2H, *p* = 0.012, 0.001)_._

## Discussion

This study examined the effect of changes in the weight of running shoes on DJ performance at three different heights. When the DJ height was lower, the PGRFT showed high side-to-side asymmetry when the participant was wearing heavy shoes. When the DJ height increased, the PGRFT was high, and the ground contact time was longer in the DJH_50_ condition.

Previous studies have shown that when the PGRF increases, the force cannot be absorbed, which leads to an increase in ACL injuries [13, 15]. A larger PGRF, early PGRFT and increased load rate will also increase lower extremity injuries [12, 20]. Early peak occurrence times and higher load rates increase the risk of injury to the lower limbs. The PGRF was greater for the DJH60 platform than for the DJH40 platform. The PGRF is proposed to increase as the height of the platform increases. Therefore, the risk of injury increases as the height of the platform increases [21]. This study found results similar to those of previous investigations, which showed that as the height of the platform increases, the PGRF and LR were increase. When PGRFT was early with higher platform, that indicates reduced shock absorption time causing the PGRF and LR increased. Thus, increasing the height of the platform increases the risk of ACL injury. Thus, increasing the height of the platform increases the risk of ACL injury.

Moreover, in this study, the ground contact time increased when the height of the platform was increased for the DJH50 conditions. This phenomenon is not beneficial for training. The DJ task induces a stretch reflex that is widely used in explosive training [22]. The reactive strength index (RSI) assesses training effectiveness, and a shorter ground contact time is associated with better explosive performance [9]. Therefore, for DJ training, a longer ground contact time will reduce the effectiveness of explosive training. One possible cause of this phenomenon is the excessive stretch stimulus caused by a high platform, which affects muscle load and muscle coordination [23]. The ground contact time was increased in this study in the DJH50 condition, resulting excessive training stimulus intensity, which reduced the training benefit of the DJ. This result is similar to those of previous studies. The ground contact time for the DJH60 platform is longer than that for the DJH20 platform [24]. Therefore, an appropriate platform height should be chosen for DJ training.

This study showed that wearing heavy shoes causes the peak impact force to occur earlier. Early occurrence of the peak impact force increases tension in the ligament and increase the risk of joint injury [25]. Therefore, in the nondominant leg, the risk of injury may be affected by shoe weight. This phenomenon was only found in the nondominant leg. The peak impact time of the dominant leg was not affected by the weight of the shoe, probably because the increased strength and flexibility of the dominant leg results in better dynamic control [14, 15]. Poor lower extremity control in the nondominant leg may lead to changes in the knee valgus angle and moment of force [26].

The RSI is an indicator used to assess the effectiveness of training. Previous studies have found that a larger RSI value within a certain range indicates a better training effect [27]. JH and ground contact time are the two major factors that affect training benefits [24, 28]. When jumping from a DJH50 or DJH60 platform, the JH will be significantly less than when a DJH20, DJH30 or DJH40 platform is used [21], and the training benefits will be reduced. Therefore, if heavy shoes are used for DJ training, in addition to causing the peak impact force of the nondominant leg to occur earlier, the reduced JH will affect the athletic performance or training effectiveness.

This study showed that an increase in platform height increases the side-to-side asymmetry of the PGRFT. Both the knee valgus moment and the knee valgus angle are significantly positively correlated with the PGRF [15]. Side-to-side asymmetry of the knee valgus angle affects the forces and moments. The strength and moment of the two feet change according to the angle of the knee joint. A larger difference between the two legs results in a higher risk of injury [14, 15, 26]. This study showed that PGRFT increases with the increase of platform height and that the rebounding action will lead to an increase in the risk of injury. Side-to-side asymmetry of the peak impact force resulting from the increased height of the platform increases the risk of injury during DJ training.

This study found that the side-to-side asymmetry of ground contact time increased when DJs are performed from a lower height. When using a lower platform (DJH20), the time from leaving the platform to first contact is not enough for subjects to achieve bilateral coordination and simultaneous landing, leading to large inter-side differences [24]. Thus, the side-to-side asymmetry in the ground contact time increases, which may lead to poor training benefits. However, no effect was observed for the DJH40 and DJH60 platforms. Therefore, the use of a lower platform for DJ tasks results in a larger side-to-side asymmetry, and both the dominant and nondominant legs should be used simultaneously for training. Additionally, this study showed that wearing a heavier shoe for the DJ increases the side-to-side asymmetry of initial ground contact time difference between legs, resulting in side-to-side asymmetry in ground contact time. This result may occur due to differences in muscle strength, flexibility and dynamic control between the dominant and nondominant legs, resulting in a difference in ground contact time [14, 15] and an increase in side-to-side asymmetry. Increased side-to-side asymmetry of the ground contact time results in poor training benefits [24]. Therefore, this study suggests that an appropriate shoe weight is used for DJ training to achieve optimal training benefits.

## Conclusion

The purpose of this study was to investigate the effects of drop height and shoe weight on ground landing impact and side-to-side asymmetry. The PGRF and LR increased when the DJ platform height increased, and the moment of PGRFT occurred later as height increased. Therefore, a higher platform results in a greater risk of injury to the lower extremities. If the height of the platform is excessive, the training stimulus will be too intense, thus reducing the training benefit of the DJ and increasing the risk of injury by increasing the side-to-side asymmetry of the peak impact force during the DJ. However, a lower platform will result in a large difference in the dominant leg and nondominant leg side of the ground contact time and initial contact time, which will affect the training benefits. Wearing heavier shoes for a DJ causes the peak impact force of the nondominant leg to occur earlier, thereby increasing the risk of injury. Additionally, wearing heavier shoes will increase the side-to-side asymmetry of the initial contact time, resulting in the increase of side-to-side asymmetry in ground contact time. Therefore, heavier shoes may affect athletic performance and training effectiveness and cause injury due to side-to-side asymmetry. The athlete may choose suitable shoe weight for DJ training to achieve optimal training benefits.

## Supporting information

S1 Dataset Experimental results of all the participants. This file contains all relative result data during experiments with the participants.
(XLSX)
